# Nitrogen fertilization coupled with foliar application of iron and molybdenum improves shade tolerance of soybean under maize-soybean intercropping

**DOI:** 10.3389/fpls.2022.1014640

**Published:** 2022-10-04

**Authors:** Jamal Nasar, Gui Yang Wang, Feng Jue Zhou, Harun Gitari, Xun Bo Zhou, Karim M. Tabl, Mohamed E. Hasan, Habib Ali, Muhammad Mohsin Waqas, Izhar Ali, Mohammad Shah Jahan

**Affiliations:** ^1^ Guangxi Key Laboratory of Agro-environment and Agro-products Safety, Guangxi Colleges and Universities Key Laboratory of Crop Cultivation and Tillage, Agricultural College of Guangxi University, Nanning, China; ^2^ Department of Agricultural Science and Technology, School of Agriculture and Enterprise Development, Kenyatta University, Nairobi, Kenya; ^3^ Agricultural Botany Department, Faculty of Agriculture (Saba Basha), Alexandria University, Alexandria, Egypt; ^4^ Bioinformatics Department, Genetic Engineering and Biotechnology Research Institute, University of Sadat City, Sadat City, Egypt; ^5^ Khwaja Fareed University of Engineering and Information Technology, Rahim, Yar Khan, Pakistan; ^6^ Department of Horticulture, Faculty of Agriculture, Sher-e-Bangla Agricultural University, Dhaka, Bangladesh

**Keywords:** abiotic stress, shade tolerance, intercropping, photosynthetic efficiencies, enzymes, growth, yield

## Abstract

Maize-soybean intercropping is practiced worldwide because of some of the anticipated advantages such as high crop yield and better utilization of resources (i.e., water, light, nutrients and land). However, the shade of the maize crop has a detrimental effect on the growth and yield of soybean under the maize-soybean intercropping system. Hence, this experiment was conducted to improve the shade tolerance of such soybean crops with optimal nitrogen (N) fertilization combined with foliar application of iron (Fe) and molybdenum (Mo). The treatments comprised five (5) maize-soybean intercropping practices: without fertilizer application (_F0_), with N fertilizer application (_F1_), with N fertilizer combined with foliar application of Fe (_F2_), with N fertilizer coupled with foliar application of Mo (_F3_) and with N fertilizer combined with foliar application of Fe and Mo (_F4_). The findings of this study showed that maize-soybean intercropping under _F4_ treatment had significantly (*p<* 0.05) increased growth indices such as leaf area (cm^2^), plant height (cm), stem diameter (mm), stem strength (g pot^-1^), and internode length (cm) and yield indices (i.e., No of pods plant^-1^, grain yield (g plant^-1^), 100-grain weight (g), and biomass dry matter (g plant^-1^)) of the soybean crop. Moreover, intercropping under _F4_ treatment enhanced the chlorophyll SPAD values by 26% and photosynthetic activities such as Pn by 30%, gs by 28%, and Tr by 28% of the soybean crops, but reduced its CO_2_ by 11%. Furthermore, maize-soybean intercropping under _F4_ treatment showed improved efficiency of leaf chlorophyll florescence parameters of soybean crops such as Fv/Fm (26%), qp (17%), ϕPSII (20%), and ETR (17%), but reduced NPQ (12%). In addition, the rubisco activity and soluble protein content of the soybean crop increased by 18% in maize-soybean intercropping under _F4_ treatment. Thus, this suggested that intercropping under optimal N fertilization combined with foliar application of Fe and Mo can improve the shade tolerance of soybean crops by regulating their chlorophyll content, photosynthetic activities, and the associated enzymes, thereby enhancing their yield and yield traits.

## 1 Introduction

Shade is the most common abiotic stress which adversely affects the plant’s growth and development when planted at higher densities such as in the greenhouse, agroforestry, and intercropping (Hussain et al., 2021; Raza et al., 2021b; Cheng et al., 2022; Nasar et al., 2022). The effect of shading leads to a change in not only enzymatic but also non-enzymatic antioxidants’ role in plants (Rajput et al., 2021). Moreover, reduced sun radiation, haze, and air pollution all contribute to a decrease in photosynthetically active radiation (Nyawade et al., 2019; Cong et al., 2020). The shaded plants adapt by lowering their photosynthetic activities and enhancing agronomic features to adjust to the diminished light quality and quantity (Hussain et al., 2021). Shade has numerous effects on plant life, resulting in diverse and novel environmental conditions. As a result, plant growth is negatively impacted, with decreased biomass, stem diameter, leaf area and thickness, stem breaking strength, and eventually yield (Hussain et al., 2019; Shafiq et al., 2020). Under intercropping, shade from one companion plant limits the photosynthesis in legumes and is considered a major threat to legume growth (Gitari et al., 2018; Nyawade et al., 2021; Blessing et al., 2022; Cheng et al., 2022).

Nitrogen is an essential component that plays a crucial part in the photosynthetic organ of a plant (Liu et al., 2018; Shao et al., 2020; Ochieng et al., 2021). Appropriate nitrogen helps in enhancing the chlorophyll content, enzymatic activity, and, enzyme content of plant leaves, hence boosting photosynthesis (Noor Shah et al., 2021). Previous studies have shown a correlation between nitrogen application rate and nitrogen utilization rate, crop photosynthetic activities, and crop production (Kong et al., 2016; Liu et al., 2019; Shah et al., 2021). According to several studies, optimal nitrogen application can effectively improve the photosynthetic properties of the plant under shading (Shah et al., 2017a; Wu et al., 2017; Raza et al., 2019). Additionally, in low-light stress situations, the combination of light and nitrogen can effectively control the photosynthetic capacity of plant leaves (Fu et al., 2017; Shah et al., 2017b). Nitrogen has also been shown to improve the chlorophyll SPAD values, photosynthetic efficiencies, and the related enzymes of soybean crops under different stress environments (Gai et al., 2017). In addition, it is also reported that maize-soybean intercropping under optimal nitrogen not only improved the growth and yield of maize crop, but also help reduce the shading effect of maize on soybean by regulating its photosynthetic and enzymatic activities (Cheng et al., 2022).

Iron (Fe) and Molybdenum (Mo) are two micronutrients that are frequently needed in smaller amounts but are crucial for plant growth and development (Togay et al., 2015). Iron is one of the key elements involved in plant chlorophyll and photosynthesis (Yoon et al., 2019). Its deficiency in plants is one of the key abiotic factors affecting the physiology and productivity of the plant (Togay et al., 2015). As earlier noted that insufficient iron reduced the number of grana and stroma lamellae per chloroplast in plant leaves (Jiang et al., 2007; Yoon et al., 2019), reducing the amount of all membrane constituents, such as electron carders of the photosynthetic electron transport chain (Wang et al., 2017; Karimi et al., 2019) and light-harvesting pigments (Wang et al., 2017). Fe deficiency also decreases the activity of ribulose 1,5 bisphosphate oxygenase/carboxylase, which is the most vital enzyme involved in plant photosynthesis (Bertamini et al., 2001; Wang et al., 2017). Previously it was reported that Fe deficiency lowers photosynthesis, photosystem II function, and rubisco activity in soybean crop (Jiang et al., 2007). In another study, maize crop was also shown to have lower chlorophyll, photosystem I and II function under Fe stress (Long et al., 2020).

On the other hand, Mo is crucial for the route that produces chlorophyll, as well as for the configuration and ultrastructure of chloroplasts, and thus plays a significant part in the photosynthetic process (Oliveira et al., 2022). The production of chlorophyll, photosynthetic efficiency, and consequently vegetative growth and grain yields are all positively associated with the configuration and ultrastructure of intact chloroplasts (Liu et al., 2018). In light of the delicate photosynthetic system, these studies suggest that Mo deficient environments might limit photosynthesis. Examples include the etiolating and yellowing of leaves (Armarego-Marriott et al., 2020), the suppression of chlorophyll biosynthesis (Wang et al., 2020), and aberrant alterations in the ultrastructure and arrangement of the chloroplast (Jin et al., 2018). Intriguingly, these traits closely resemble N deprivation, which has been linked to yellowing of leaves, decreased chlorophyll concentrations, and irregularly shaped or almost circular chloroplasts, according to other investigations (Liu et al., 2018). Foliar application of molybdenum has also been shown to improve the photosynthetic activities and nitrogen assimilatory enzymes of maize and soybean in maize-soybean intercropping, thereby enhancing their growth and yield (Oliveira et al., 2022). Therefore, it is possible to hypothesize that Mo, in addition to improving chloroplast structure and chlorophyll synthesis, may also enhance photosynthesis through effective N uptake and absorption.

In China, various cropping systems account for over half of the overall grain yield (Xu et al., 2020). Among these, intercropping of maize and soybeans has significantly increased soybean productivity. Approximately 667 thousand hectares of land in southwest China are used for maize-soybean intercropping, and the area is still expanding as a result of the rising demand for foods high in protein (Raza et al., 2020). However, the lodging of soybean seedlings is a significant issue in such intercropping system given that maize overshadows soybeans throughout the co-growth stage, which resulted in lower component yield besides being incompatible with mechanization (Chang et al., 2020). Consequently, this makes it hard to meet the need for high efficiency and higher yields in modern agriculture. Numerous studies have shown that stem properties like morphology, physiology, and biomechanics are closely associated with lodging resistance with the main stem strength being the most important factor in enhancing the lodging resistance of soybean. (Liu et al., 2018; Liu et al., 2019). To counteract the negative effects of shade, several practices have been put in place to optimize plant growth, which include the use of plant growth regulators (Sabagh et al., 2021), shade tolerant cultivars (Paradiso and Proietti, 2022), appropriate ${\text{NH}}_{4}^{+}:{\text{NO}}_{3}^{-}$ ratio (Raza et al., 2021a) and titanium application (Hussain et al., 2021). Nonetheless, to the best of our knowledge, the physiological and agronomic activities of soybean under shade stress specifically in maize-soybean intercropping under the nitrogen application combined with foliar use of micronutrients (i.e., Fe and Mo) are unclear.

Therefore, this study was designed to examine the effects of nitrogen fertilization in combination with foliar applications of Fe and Mo on the growth and production of soybean under intercropping environments. The main objective of this study was to promote and improve the growth, yield, and photosynthesis system of soybean crop with optimal N fertilization combined with foliar application of Fe and Mo under the shading environment of intercropping.

## 2 Materials and methods

The current study was conducted in the late summer growing season from September 2021 to February 2022 at Guangxi University’s research center in Nanning, China. With an average annual rainfall of 1080 mm, this region has a subtropical monsoon climate. The physio-biochemical properties of experimental soil showed that soil had a loam texture, 23.7 g kg^-1^ of organic matter, 0.118 percent total nitrogen, 109.9 mg kg^-1^ of alkaline nitrogen, and a pH of 7.4. In addition, it had 74.0, 73.6, 97.7 and 0.1 mg kg^-1^ of available potassium, phosphorus, iron and molybdenum, respectively.

**Figure 1:**
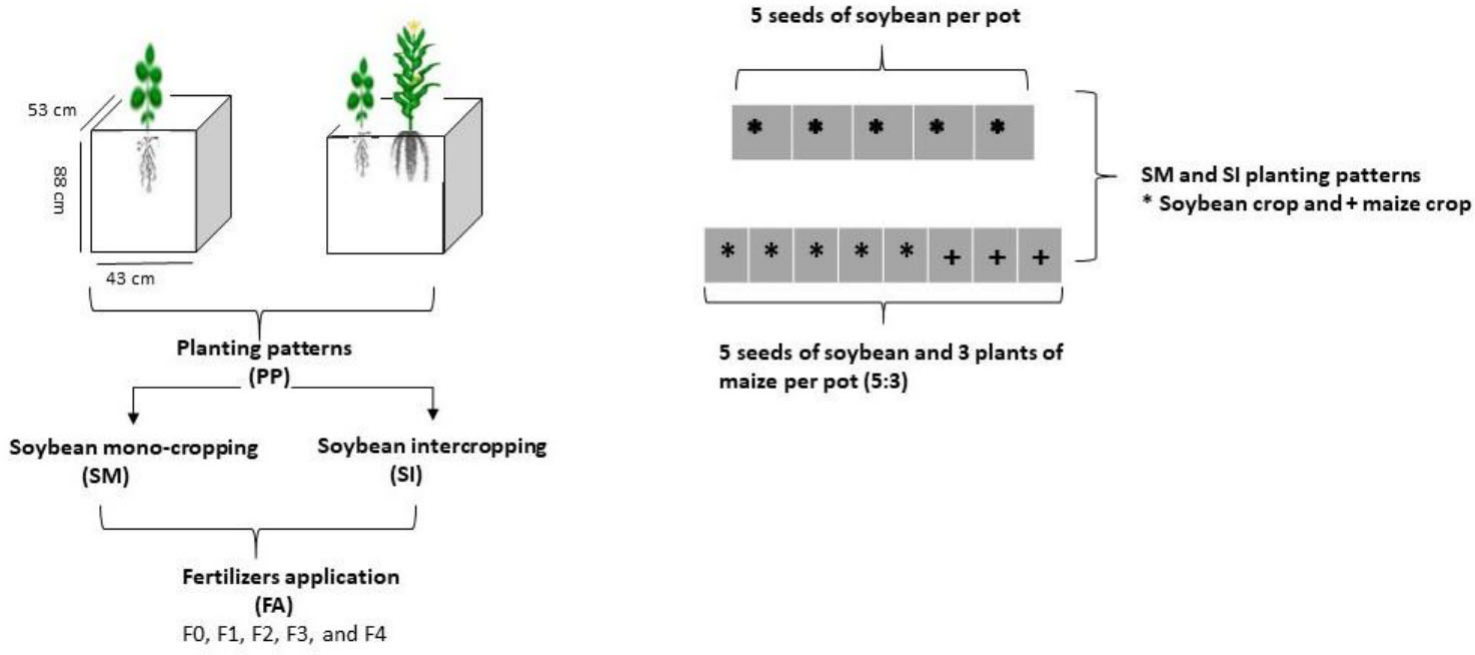
Schematic diagram of the experiment. _F0_; without fertilizer application, _F1_; nitrogen fertilizer application, _F2_; nitrogen fertilizer with foliar application of iron, _F3_; nitrogen fertilizer with foliar application of molybdenum, _F4_; nitrogen fertilizer with foliar application of iron and molybdenum).

**Figure 2:**
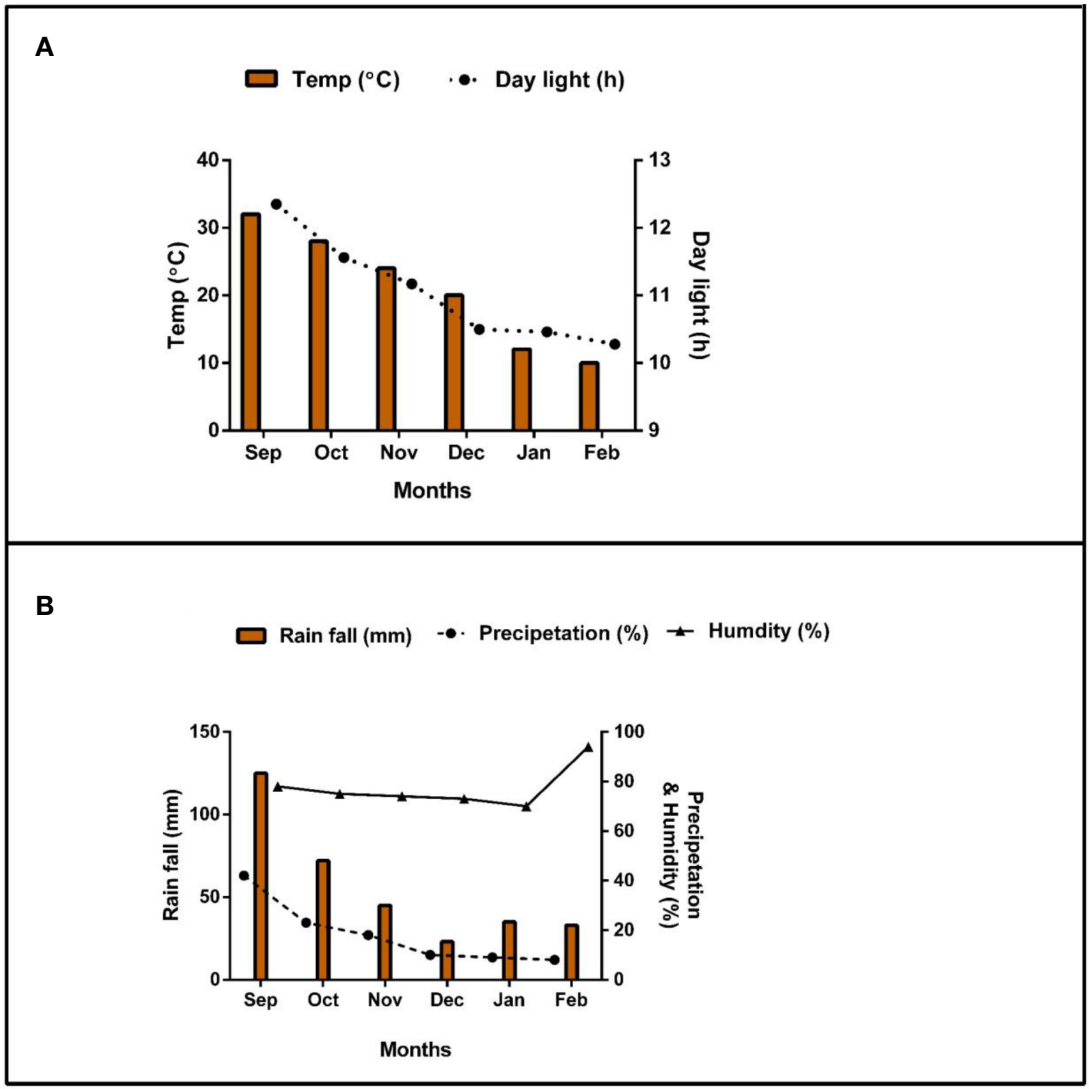
Weather forecast [**(A)**; temperature and daylight hours and **(B)**; rainfall, precipitation, and humidity] report of the experimental area during the experiment period.

Soybean crop (Gui Chun 15 variety) was planted under mono-cropping (SM) and intercropping (SI) with maize (Ching Ching 700 variety) in pots (i.e., 88 cm height, 53 cm width, and 43 cm length) (**Figure 1**). Initially, 10 seeds of soybean were sown in mono-cropping and in intercropping with 5 plants of maize at a field plant density of 20 kg soybean seeds ha^-1^ and 60,000 maize plants ha^-1^, respectively. However, at the V3 growth stage, the soybean plants were reduced to 5 and maize plants to 3 (5:3) in each pot to better adapt to the pot environment. Each treatment pot was filled with 120 kg of soil, replicated four times, and randomly placed (Completely randomized design CRD) in a ventilated net house under natural light environment. Plants were sown in the mid of September (2021) and harvested in mid of February (2022). For fertilizer applied treatments, nitrogen fertilizer (@ 100 kg ha^-1^) was applied before sowing by mixing it with the experimental soil. The foliar application of iron @ 0.15 mg g^-1^ and molybdenum @ 0.10 mg g^-1^ was carried out in three splits: at the V5, R1, and R5 stages. Nevertheless, phosphorous and potassium were applied uniformly to all treatment pots (i.e., P at 100 kg ha^-1^ and K at 50 kg ha^-1^). Nitrogen (N) was applied in form of urea (46% N), phosphorus as diammonium phosphate (P_2_O_5_ 46% P), potassium as potassium chloride (K_2_O 60% K), iron as ferrous sulphate (FeSO_4_ 20.5% Fe), and molybdenum as an ammonium molybdate ((NH_4_)_6_Mo_7_O_24_ 54% Mo). The treatments included five (5) maize-soybean practices: without fertilization (_F0_), nitrogen fertilizer (_F1_), nitrogen fertilizer coupled with foliar application of Fe (_F2_), nitrogen fertilizer coupled with foliar application of Mo (_F3_), and nitrogen fertilizer coupled with foliar application of Fe and Mo (_F4_). Different agronomic practices such as irrigation and control of weeds and insect pests were carefully monitored. Meteorological parameters such as temperature (°C), precipitation (%), rainfall (mm), daylight (hrs), and humidity (%) were recorded throughout the experiment and presented in **Figure 2**.

### 2.1 Data collection

#### 2.1.1 Growth indices

The growth indices such as stem diameter, plant height, stem strength, internode length, and leaf area of soybean crops were determined at V5 and R5 (Hussain et al., 2021). Plant height was measured from top to bottom using a measuring tape, and stem diameter with a Vernier caliper whereas internode length and stem strength were measured using a digital force tester (YYD-1, Zhejiang Top Instrument Hangzhou, China). The leaf area was measured with LI -3000C portable leaf area meter (LI-COR, Nebraska, USA).

#### 2.1.2 Yield indices and biomass dry matter

The yield indices and biomass dry matter of soybean crops were obtained at full maturity when harvesting was done (Raza et al., 2020). The pods were counted and recorded per plant after which they were removed from the plant and threshed to determine 100 seeds’ weight and grain yield by weighing on an electric scale. After threshing, the remaining plant straw was sun-dried and oven-dried at 65°C for 72 h to obtain dry biomass matter.

#### 2.1.3 Chlorophyll SPAD and photosynthetic activities

Chlorophyll SPAD values and photosynthetic activities such as photosynthetic rate (Pn) (µmol CO_2_ m^-2^ s^-1^), stomatal conductance (gs) (mol H_2_O m^-2^ s^-1^), intercellular CO_2_ (Ci) (mol CO_2_ mol^-1^), and transpiration rate (Tr) (µmol H_2_O m^-2^ s^-1^) of soybean crop were measured at the V5 stage (Hussain et al., 2021). These indices were measured on a bright sunny day in the morning (between 9:00 am and 11:00 am) at a fully expanded leaf (usually 3 measurements per leaf) from the healthiest plant. The chlorophyll content was estimated using the SPAD Chlorophyll Meter (SPAD-502, Minolta Camera, Tokyo, Japan) while photosynthetic activities were measured using the Li-6400XT portable photosynthesis system (Licor Inc., Lincoln, NE, USA) at an adjusted constant light of 80, 100, 150, 200, 400, 600, 800, and 1000 μmol m^-2^ s^-1^ and a leaf temperature of ~27°C with a continuous CO_2_ level of 400 μmol mol^-1^ as per procedures provided by Ahmad et al. (2022a).

#### 2.1.4 Chlorophyll fluorescence parameters

Chlorophyll Fluorescence parameters such as maximum PSII quantum yield (Fv/Fm), actual PSII efficiency (ϕPSII), photochemical quenching coefficient (qp), non-photochemical quenching coefficient (NPQ), and the electron transport rate (ETR) of soybean crop were measured at night (full dark) on the corresponding day when chlorophyll and photosynthetic activities were measured (Iqbal et al., 2019). These indices were measured using the same portable photosynthesis system used for the estimation of photosynthetic activities at a leaf temperature of ~27°C by adjusting the system according to provided manual for dark.

#### 2.1.5 Rubisco activity

Rubisco enzyme activity in soybean leaves was measured at the V5 stage using a Rubisco enzyme ELISA kit (96 micropores) sourced from Shanghai Fu Life Industry Co. Ltd., Shanghai, China. In brief, 1 g of frozen leaf samples were minced using a mortar and pestle and an icebox using 2 ml of 50 mmol L^−1^ phosphate buffer solution (pH 7.8). For 15 min, the solution was centrifuged at 7000 rcf at 4°C after which the level of plant Rubisco activase was determined by employing a double antibody sandwich method. In this case, the microspore plate encapsulated the Rubisco activase antibody forming a solid phase antibody and then adding to the microspore of the monoclonal antibody. Firstly, 10 ml of sample solution was added to the micropore plate followed by the addition of 40 µl of phosphate buffer solution as a sample diluent. The micropore plate was kept incubated for 30 min at 37°C with a plastic sheet covering it and this incubation underwent five rounds. The 3,3´5,5´-tetramethylbenzidine was transferred with the help of the enzyme horseradish peroxidase, which was initially colored blue before changing to yellow when subjected to acid. Within 15 minutes after administering the stop solution, an enzyme marker used a 450 nm wavelength to quantify the absorbance. The sample’s RA was calculated using a standard curve and represented as U g_-1_ (Ali et al., 2022).

#### 2.1.6 Total soluble protein

Soybean leaf tissues were used to determine the soluble protein. In brief, 3.0 g of fresh leaf tissue were homogenized in 9 ml of 0.1 M Tris-HCl buffered at pH 8.0 and extracted at a constant temperature of 4°C. The extract was centrifuged at 12,000 revolutions per minute for 30 minutes, and the supernatant was utilized to make a basic enzyme extract. Protein content was assessed following trichloroacetic acid precipitation using bovine serum albumin as a standard. The soluble protein-containing supernatants were heated to 100°C for 10 minutes, then placed on ice before being centrifuged at maximum speed (15 000 g) in a microcentrifuge for 15 minutes at 4°C to obtain the fraction of termostable soluble proteins. The electrophoresis on 7.5% polyacrylamide gel (PAG) was used to separate total and termostable soluble proteins. The obtained total soluble protein was expressed in U g_-1_ (Kirova et al., 2005).

#### 2.1.7 Land Equivalent Ratio

The land equivalent ratio (LER) was computed as indicated in Equation 1.


(1) $$\text{LER}=\left(\frac{{\text{Y}}_{\text{im}}}{{\text{Y}}_{\text{mm}}}+\frac{{\text{Y}}_{\text{si}}}{{\text{Y}}_{\text{sm}}}\right)\;$$

Where Yim and Ysi exemplify the grain yield of maize and soybean crops in intercropping whereas Ymm and Ysm represent the respective yields under mono-cropping. The LER is an indicator used to determine the competitiveness between intercrops for the utilization of the available resources (Gitari et al., 2020; Maitra et al., 2020). If the value of LER is 1, indicates that both monocrop and intercrop produce equal yield and utilize the available resources equally. If the value of the LER is greater than 1, suggests a greater complementary effect of intercropping maize than a competitive one, and produces a higher yield compared to mono-cropping. If the value of LER is less than 1, indicates interspecific competition is greater than interspecific facilitation, and there is no intercropping advantage. So, the higher the LER, the greater the benefit of increasing yield in intercropping over mono-cropping (Soratto et al., 2022).

### 2.2 Data analysis

The data were computed and formulated in Ms-Excel 2016 and statistically analyzed using the statistical analysis software ms-statistix 8.1. A two-way factorial ANOVA was performed to test the significance level (*p* < 0.05) between means at LSD test by the fertilizer application (FA) and the planting patterns (PP) factors as well as their interactions. Graphical and statistical software (Graphpad Prism 6.1) was used to construct graphs. The linear regression was used to determine the relationship between the photosynthetic rate (Pn) with chlorophyll SPAD values, leaf area, grain yield, biomass dry matter, and other photosynthetic activities (i.e., Tr, Co_2_ and gs). The relationship of Pn was also tested with leaf chlorophyll florescence parameters (i.e., Fv/Fm, ϕPSII, qp, NPQ and ETR), Rubisco activity, and soluble protein of the soybean crop (Ahmad et al., 2022b).

## 3 Results

### 3.1 Growth indices

Intercropping without fertilizer application showed lower trends in the growth indices of soybean crops such as stem diameter (mm), plant height (cm), stem strength (g pot^-1^), and leaf area (cm^2^) (**Table 1**). However, with fertilizer application, intercropping showed significant (*p*< 0.05) improvement in these indices of the soybean crop. Under intercropping, the soybean plant height was 57, 62, 60, 66, and 79 cm in _F0_, _F1_, _F2_, _F3_, and _F4_ treatment, respectively whereas the respective stem diameters were 11, 12, 13, 12, and 14 mm. The stem strength for these plants was noted as 362 g pot^-1^ (_F0_), 376 g pot^-1^ (_F1_), 394 g pot^-1^ (_F2_), 388 g pot^-1^ (_F3_), and 426 g pot^-1^ with respective leaf areas of 255, 270, 287, 285 and 302 cm^2^. Nevertheless, it was observed that intercropping with fertilizer application reduced the internode length of the soybean by 9, 6, 4, 5, and 2% in _F0_, _F1_, _F2_, _F3_, and _F4_ treatment, respectively.

**Table 1:** Growth indices of soybean crop as influenced by different fertilizer application and planting patterns.

Fertilizer application (FA)	Planting pattern (PP)	Plant height (cm)	Leaf area (cm^2^)	Stem diameter (mm)	Stem strength (g pot^-1^)	Internode length (cm)
_F0_	SM	54.15 ± 8.0 c	246.65 ± 12.8 e	10.40 ± 0.1 f	345.05 ± 4.3 g	11.15 ± 1.1 bc
	SI	65.95 ± 8.5 bc	255.20 ± 12.5 de	10.67 ± 0.2 f	362.48 ± 3.1 d	10.20 ± 1.0 c
_F1_	SM	57.20 ± 7.7 bc	250.95 ± 10.7 de	11.07 ± 0.3 e	351.92 ± 5.1 gf	12.20 ± 1.2 ab
	SI	62.35 ± 8.9 bc	270.35 ± 9.7 c	11.75 ± 0.3 d	375.93 ± 4.1 c	11.50 ± 1.1 b
_F2_	SM	60.02 ± 8.1 bc	252.52 ± 9.9 de	11.70 ± 0.3 d	354.70 ± 4.1 ef	12.40 ± 1.0 ab
	SI	67.80 ± 6.6 ab	286.80 ± 4.5 b	12.65 ± 0.2 b	393.95 ± 5.8 b	11.90 ± 1.1 ab
_F3_	SM	59.15 ± 8.1 bc	256.15 ± 7.4 de	11.62 ± 0.1 d	356.98 ± 3.5 ef	12.37 ± 0.8 ab
	SI	65.72 ± 7.2 bc	285.23 ± 3.6 b	12.15 ± 0.2 c	387. 92 ± 5.2 b	11.90 ± 0.8 ab
_F4_	SM	63.95 ± 9.5 bc	261.70 ± 10.1 cd	11.95 ± 0.2 cd	367.17 ± 8.1 cd	12. 65 ± 1.0 a
	SI	79.02 ± 13.8 a	302.03 ± 9.4 a	13.57 ± 0.3 a	426.45 ± 13.5 a	12.35 ± 1.0 ab
**Significance levels**						
**FA**		0.01***	0.00***	0.00***	0.00***	0.01***
**PP**		0.01***	0.00***	0.00***	0.00***	0.04***
**FA×PP**		0.70^ns^	0.02^***^	0.00***	0.00***	0.97^ns^

The means with ± standard deviations (SD) having different lower-case letters (down the column) differ significantly at the LSD test p ≤ 0.05 level of probability. FA; fertilizer application regime, PP; planting patterns, _F0_; no fertilization, _F1_; nitrogen fertilization, _F2_; nitrogen fertilization combined with the foliar application of Fe, _F3_; nitrogen fertilization combined with foliar application of Mo, _F4_; nitrogen fertilization combined with foliar application of Fe and Mo. ***p ≤ 0.001, ^ns^p > 0.05.

### 3.2 Yield indices and biomass dry matter

Shading of soybean by maize under maize-soybean intercropping without fertilizer applications resulted in a significant (*p*< 0.05) decrease in the yield indices and biomass dry matter (g plant^-1^) of the soybean crop (**Table 2**). However, the indices increased when intercropping was practiced with fertilizer application. For instance, an 8% reduction in pods plant^-1^ was noted in _F0_ compared to increases of 15, 13, 11, and 22% that were noted for _F1_, _F2_, _F3_, and _F4_, respectively. Similarly, compared with _F1_, _F2_, _F3_, and _F4_ in which 10, 7, 5, and 16% increases in grain yield (g plant^-1^) were noted, respectively, the yield in _F0_ decreased by 13%. A comparable trend was noted for 100 seeds weight (g), with a decrease of 7% in _F0_ and increases of 12, 8, 7, and 19% in _F1_, _F2_, _F3_, and _F4_, respectively. Similarly, for biomass dry matter (g plant^-1^), with exception of _F0_ where a decrease of 11% was recorded, intercropping with fertilizer resulted in 10, 9, 6, and 17% increase in _F1_, _F2_, _F3_, and _F4_, respectively. With exception of F4 which had a land equivalent ratio greater than one (1.03), the other treatments recorded values below one ranging from 0.86 in F0 to 0.96 in F2.

**Table 2:** Yield indices and biomass dry matter of soybean crop as influenced by different fertilizer application and planting patterns.

Fertilizer application (FA)	Planting pattern (PP)	No of pods plant^-1^	Grain yield (g plant^-1^)	100-grain weight (g)	Biomass dry matter (g plant^-1^)	LER
_F0_	SM	27.50 ± 1.3 fg	72.57 ± 5.3 d	14.85 ± 0.5 f	82.85 ± 5.8 e	
	SI	25.75 ± 1.2 g	62.97 ± 5.9 e	13.85 ± 1.0 g	74.15 ± 3.1 f	0.86
_F1_	SM	30.75 ± 1.7 de	74.12 ± 4.5 cd	16.32 ± 0.4 de	90.40 ± 4.1 cd	
	SI	35.50 ± 1.3 bc	81.35 ± 4.7 b	18.25 ± 1.3 b	99.33 ± 3.3 b	0.91
_F2_	SM	29.00 ± 1.1 ef	75.72 ± 5.6 bcd	15.87 ± 0.3 e	86.55 ± 4.5 de	
	SI	32.75 ± 1.7 cd	80.65 ± 2.3 b	17.07 ± 0.5 cd	93.67 ± 3.5 bc	0.96
_F3_	SM	27.75 ± 1.5 fg	76.35 ± 3.3 bcd	16.37 ± 0.4 de	84.85 ± 4.1 de	
	SI	30.75 ± 1.7 de	79.90 ± 3.4 bc	17.52 ± 0.2 bc	90. 00 ± 5.1 cd	0.94
_F4_	SM	36.00 ± 1.8 b	80.95 ± 3.8 b	17.67 ± 1.2 bc	94.75 ± 6.4 bc	
	SI	43.75 ± 4.2 a	93.67 ± 2.5 a	20.95 ± 1.4 a	110.75 ± 2.4 a	1.03
**Significance levels**						
**FA**		0.00***	0.00***	0.00***	0.00***	
**PP**		0.00***	0.01**	0.00***	0.00***	
**FA×PP**		0.00***	0.00***	0.00***	0.00***	

The means with ± standard deviations (SD) having different lower-case letters (down the column) differ significantly at the LSD test p ≤ 0.05 level of probability. FA; fertilizer application, PP; planting patterns, _F0_; no fertilization, _F1_; nitrogen fertilization, _F2_; nitrogen fertilization combined with foliar application of Fe, _F3_; nitrogen fertilization combined with foliar application of Mo, _F4_; nitrogen fertilization combined with foliar application of Fe and Mo. ***p ≤ 0.001, **p < 0.01.

### 3.3 Chlorophyll content and photosynthetic activities

**Figure 3:**
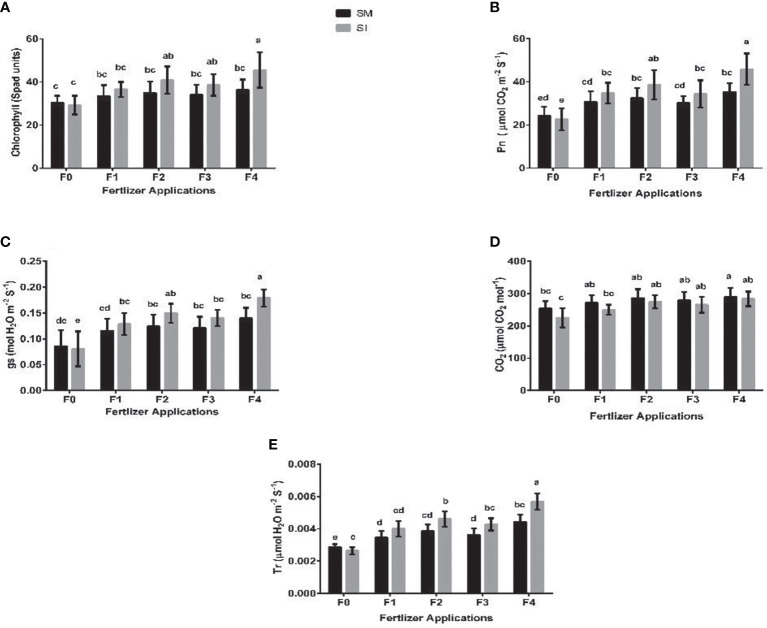
Chlorophyll SPAD values **(A)**, Pn **(B)**; gs **(C)**, CO_2_ **(D)**, and Tr **(E)** of soybean crop under different planting patterns such as SM; soybean mono-cropping, SI; soybean intercropping and fertilizer application treatments such as _F0_; without fertilizer application, _F1_; nitrogen fertilizer application, _F2_; nitrogen fertilizer with foliar application of iron, _F3_; nitrogen fertilizer with foliar application of molybdenum, _F4_; nitrogen fertilizer with foliar application of iron and molybdenum). Pn; photosynthetic rate, gs; stomatal conductance, CO_2_; intercellular carbon dioxide and Tr; transpiration rate). The column bars with dissimilar lowercase letters are significantly different from each other as per the LSD test (p< 0.05).

Intercropping without fertilizer application reduced the chlorophyll and photosynthetic activities of the soybean crop, but these indices increased significantly (*p*< 0.05) though marginally when intercropping was practiced with fertilizer application (**Figure 3**). For instance, under _F0_, intercropping resulted in reduced chlorophyll SPAD values of soybean crop by 4%, and increases of 9, 17, 14, and 26% in _F1_, _F2_, _F3_, and _F4_ treatment, respectively. Similarly, intercropping decreased the Pn (µmol CO_2_ m^-2^ s^-1^) of soybean by 7% in _F0_, but increased it by 13, 19, 14, and 30% in _F1_, _F2_, _F3_, and _F4_ treatment, respectively. Moreover, despite gs (mol H_2_O m^-2^ s^-1^) increasing by 11, 20, 16, and 28% in _F1_, _F2_, _F3_, and _F4_ treatment, respectively, a decrease of 6% was noted in the _F0_ treatment. Likewise, intercropping decreased the Tr (µmol H_2_O m^-2^ s^-1^) by 9% in _F0_, with increases of 16, 20, 18, and 28% being recorded for _F1_, _F2_, _F3_, and _F4_, respectively. In contrast, intercropping reduced the CO_2_ (mol CO_2_ mol^-1^) of soybean in all treatments in a decreasing order of 11% (_F0_)< 8% (_F1_)< 5% (_F2_)< 4% (_F3_)< 2% (_F4_).

### 3.4 Chlorophyll fluorescence

**Figure 4:**
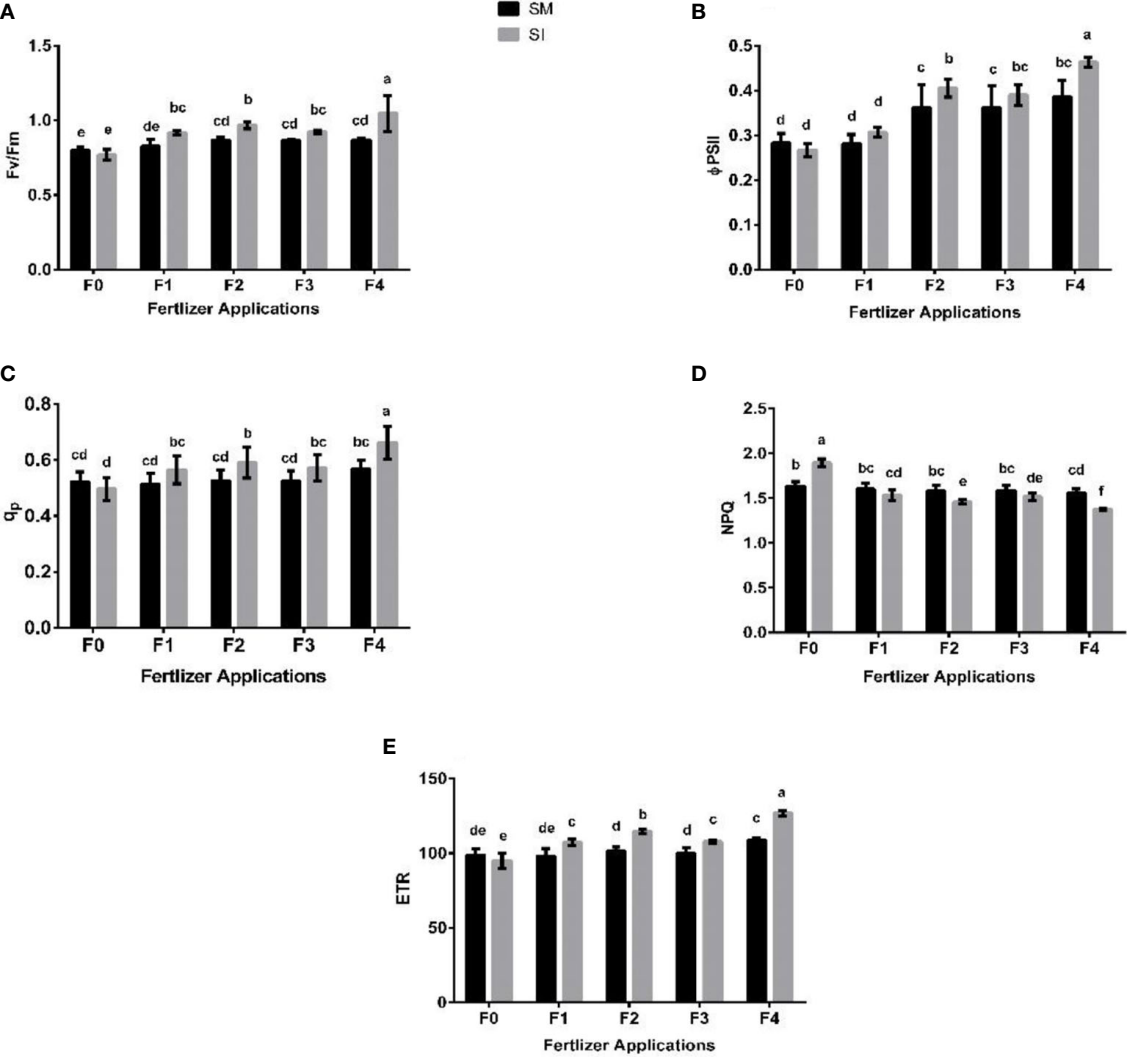
Leaf chlorophyll fluorescence parameters such as Fv/Fm; maximum fluorescence **(A)**, ϕPSII; efficiency of the photosystem **(B)**, qp; photochemical quenching **(C)**, NPQ; non-photochemical quenching **(D)**, ETR; electron transport rate **(E)** of soybean crop under different planting patterns such as SM; soybean mono-cropping, SI; soybean intercropping and fertilizer application treatments such as _F0_; without fertilizer application, _F1_; nitrogen fertilizer application, _F2_; nitrogen fertilizer with foliar application of iron, _F3_; nitrogen fertilizer with foliar application of molybdenum, _F4_; nitrogen fertilizer with foliar application of iron and molybdenum. The column bars with dissimilar lowercase letters are significantly different from each other as per the LSD test (p< 0.05).

The chlorophyll fluorescence parameters (i.e., Fv/Fm, qp, ϕPSII, ETR, and NPQ) varied significantly (*p*< 0.05) among the intercropping systems and fertilizer application regimes (**Figure 4**). That is, they decreased under intercropping without fertilizer application but increased with fertilizer application. In _F0_ treatment, a decrease of 4% was recorded for Fv/Fm whereas increases of 11, 12, 7, and 21% were noted in _F1_, _F2_, _F3_, and _F4_ treatment, respectively. Based on the qp, intercropping without fertilizer application (_F0_) resulted in a 5% decrease as opposed to increases of 10, 12, 9, and 17% that were noted in treatments that received fertilizer, i.e. _F1_, _F2_, _F3_, and _F4_, respectively. Congruently, there were respective increases in ϕPSII of 9, 12, 8, and 20% in comparison with a 6% reduction for _F0_. In addition, intercropping without fertilizer application (_F0_) resulted in a 4% decrease in ETR whereas with the integration of fertilizer, the parameter increased by 9, 13, 8, and 17% in _F1_, _F2_, _F3_, and _F4_, respectively. In contrast, NPQ increased by 16% in _F0_, and decreased under fertilizer application by 4% (_F1_), 8% (_F2_), 5% (_F3_) and 12% (_F4_).

### 3.5 Rubisco activity and soluble protein

**Figure 5:**
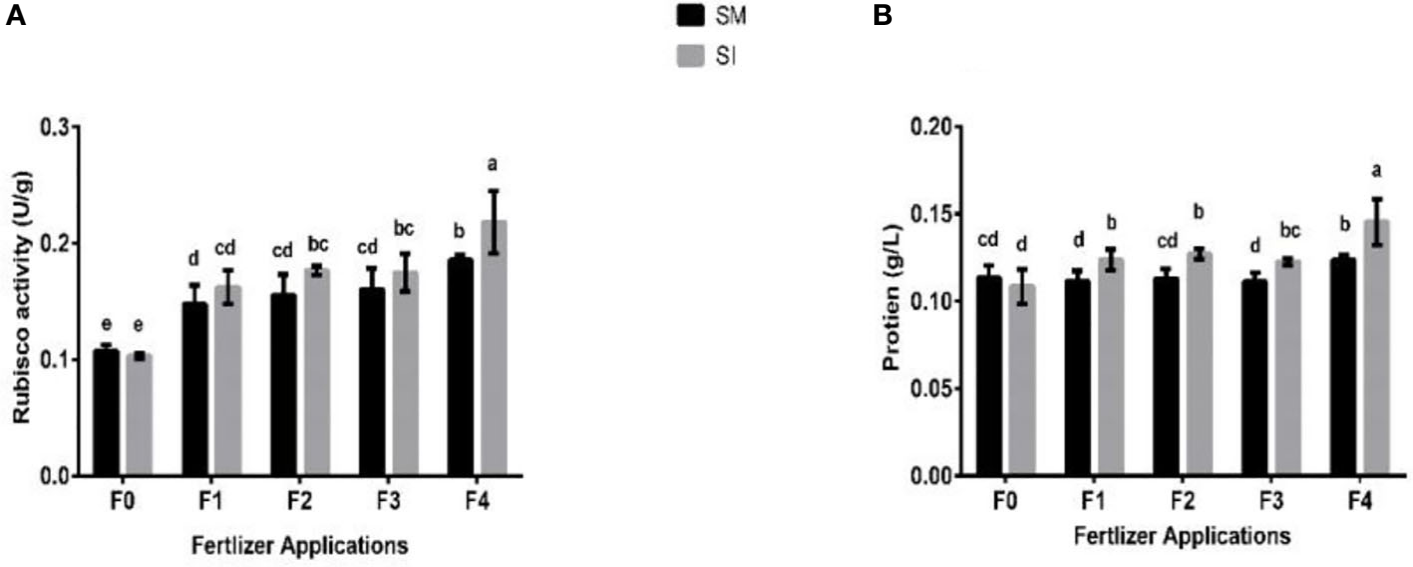
Rubisco activity **(A)** and soluble protein **(B)** of soybean crop under different planting patterns such as SM; soybean mono-cropping, SI; soybean intercropping and fertilizer application treatments such as _F0_; without fertilizer application, _F1_; nitrogen fertilizer application, _F2_; nitrogen fertilizer with foliar application of iron, _F3_; nitrogen fertilizer with foliar application of molybdenum, _F4_; nitrogen fertilizer with foliar application of iron and molybdenum. The column bars with dissimilar lowercase letters are significantly different from each other as per the LSD test (p< 0.05).

Maize-soybean intercropping practiced without fertilizer application resulted in significant (*p*< 0.05) lower rubisco activity (U g_-1_ plant^-1^) and soluble protein (U g_-1_ plant^-1^) of soybean crop compared to when the crops were boosted with fertilizer (**Figure 5**). For instance, the rubisco activity in _F0_ was reduced by 4% and increased by 10% in _F1_, 14% in _F2_, 9% in _F3_, and 18% in _F4_. Similarly, there was a 4% decrease in the soluble protein of soybean in _F0_ treatment, and increases of 11, 13, 10, and 18% in _F1_, _F2_, _F3_, and _F4_, respectively.

### 3.6 Liner regression

**Figure 6:**
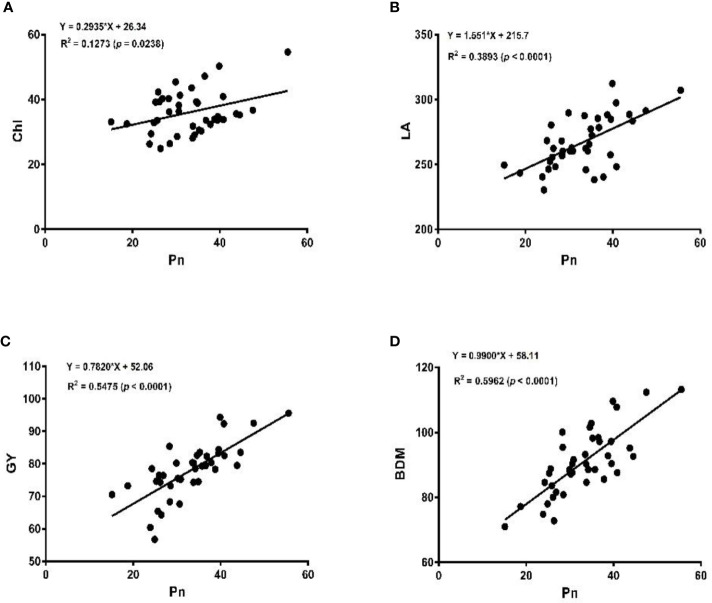
Linear regression of photosynthetic rate (Pn), with chlorophyll content (Chl) **(A)**, leaf area (LA) **(B)**, grain yield (GY) **(C)**, and biomass dry matter (BDM) **(D)** of the soybean crop.

**Figure 7:**
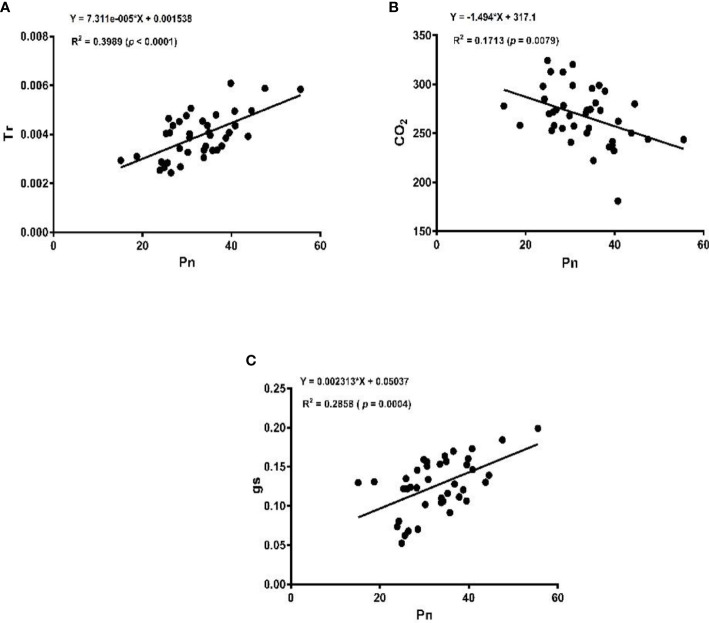
Linear regression of photosynthetic rate (Pn) with transpiration rate (Tr) **(A)**, intercellular carbon dioxide (CO_2_) **(B)**, and stomatal conductance (gs) **(C)**.

**Figure 8:**
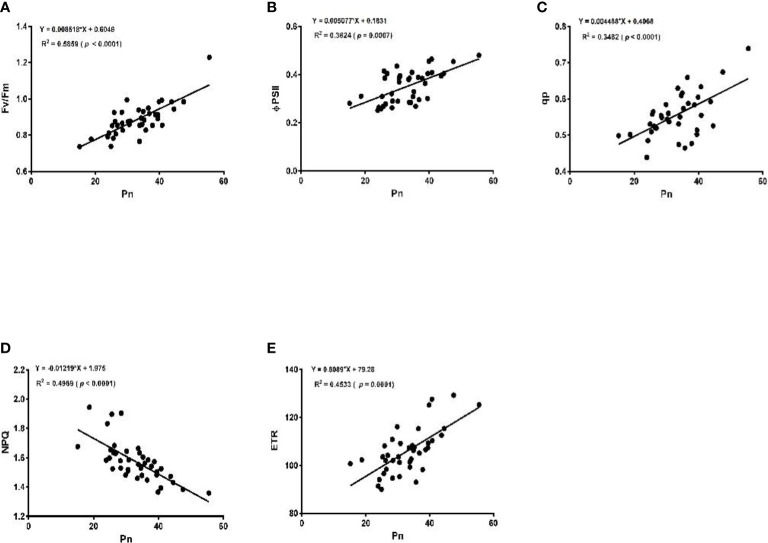
Linear regression of photosynthetic rate (Pn) with maximum fluorescence (Fv/Fm) **(A)**, efficiency of photosystem (ϕPSII) **(B)**, photochemical quenching (qp) **(C)**, non-photochemical quenching (NPQ) **(D)**, and electron transport rate (ETR) **(E)**.

**Figure 9:**
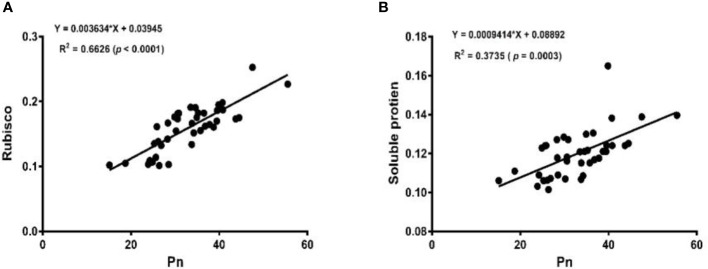
Linear regression of Pn with Rubisco activity **(A)** and soluble protein **(B)** of soybean crop.

The linear regression analysis showed that Pn had strong correlations with chlorophyll, LA, grain yield, and biomass dry matter (**Figure 6**). With exception of NPQ and CO_2_ where Pn had negative correlations, it indicated significant and strong associations with Tr, gs, rubisco, soluble protein, and leaf chlorophyll fluorescence parameters such as Fv/Fm, ϕPSII, qp, and ETR (**Figures 7**–**9**).

## 4 Discussion

Shade is the most common abiotic stress, which adversely affects the physiological and agronomic traits of plants. Under a maize-soybean intercropping environment, the shading of maize crops restricts the growth of soybean crop mainly by changing the direction or blocking the direct solar radiation, which in turn reduce the chlorophyll, photosynthetic activities, growth indices, enzymes, and ultimately its yield (Jiao et al., 2013; Hussain et al., 2021). However, these negative effects of shading can be effectively reduced with optimal fertilization of nutrient elements particularly N, Fe, and Mo. Nitrogen is an important element for plant growth and devolvement, because of its direct involvement in the plant amino acid (the building block of plant protein), nucleic acid (forms plant DNA), and photosynthetic carbohydrates formation (Chen et al., 2017). Iron on the other side plays an important role in plant chlorophyll and photosynthesis, which gives plant oxygen and healthy green color (Wang et al., 2017; Nasar et al., 2022). This is why iron deficient plant shows chlorosis or a silky yellow color on their leaves, thus making iron a crucial element for plant growth and development. Molybdenum (Mo) is an essential micronutrient for plant photosynthetic process because of its key role in the chlorophyll biosynthesis pathway, chloroplast ultrastructure, and configuration (Togay et al., 2015; Imran et al., 2019). It also forms an important component of nitrate reductase, nitrogenase, and leghemoglobin, which improve the nutritional value of the crop resulting in better growth and yield production (Wang et al., 2017; Oliveira et al., 2022). Previous studies have focused on the effect of N, Fe, and Mo on the growth and yield of soybean crops (Gai et al., 2017; Gülser et al., 2019; Oliveira et al., 2022). However, the combined effect of these elements on the soybean crop under intercropping environment has not been reported so far.

The present study demonstrated that maize-soybean intercropping without fertilization (treatment _F0_) resulted in weak physio-agronomic efficiency of the soybean crop. However, these indices were improved under fertilizer application treatments (i.e., _F1_, _F2_, _F3_, and _F4_), but the _F4_ treatment showed promising results. The low agronomic traits of the soybean crop in the intercropping under _F0_ treatment could be attributed to the strong shading effect of the maize crop as reported earlier (Hussain et al., 2021; Cheng et al., 2022). However, the improvement in these traits in the intercropping under other treatments (i.e., _F1_, _F2_, _F3_, and _F4_) could be linked to the application of N, Fe, and Mo, which help plants retain their growth under stress environment (Gai et al., 2017; Gülser et al., 2019; Seleiman et al., 2021; Mirriam et al., 2022; Oliveira et al., 2022). As previously reported, shading of maize in maize-soybean intercropping had negatively affected the canopy structure and stem characteristics, which resulted in lower physio-agronomic performances of soybean crop (Cheng et al., 2022). In another study, the lower component yield and weak growth of soybean crop were mainly because of soybean lodging due to the shade of maize in maize-soybean intercropping (Hussain et al., 2021). However, soybean retains its growth and yield under different environmental stresses when fertilized with optimal fertilizer application of N, P, Fe, and Mo (Gai et al., 2017; Gülser et al., 2019; Seleiman et al., 2021; Mirriam et al., 2022; Oliveira et al., 2022).

The leaf area is the key source of carbon assimilation and light interception while chlorophyll is an important pigment involved in not only the absorbance of solar energy but also in its transmission and conversion into electrochemical energy. The current study reported low leaf area and decreased SPAD values of the soybean crop in intercropping under _F0_ treatments, which could be ascribed to increased shade under stress (Wu et al., 2017). However, under fertilizer application (i.e., _F1_, _F2_, _F3_, and _F4_) intercropping significantly increased the leaf area and SPAD values of the soybean crop, but the _F4_ treatment was more evident. The increase in leaf area is mainly linked with higher chlorophyll content and net photosynthetic rate. Equally, the study demonstrated a significant improvement in the photosynthetic activities (i.e., Pn, gs, and Tr) of the soybean crop in the intercropping at _F1_, _F2_, _F3_, and _F4_ treatment as compared to _F0_ treatment. These results are supported by the previous reports of Razaq et al. (2017), who stated that the shade of maize in maize-soybean intercropping causes a significant decrease in the chlorophyll SPAD, photosynthetic activities, and ultimately its yield, but under nitrogen fertilization, these indices improved to a certain extent. Iron foliar application is also shown to improve the photosystem I and photosystem II function of soybean crop under different stress environments (Jiang et al., 2007), which confirmed our results. In another study, molybdenum foliar application has been shown to improve the shade tolerance of soybean crop by regulating its chlorophyll, photosynthetic, and rubisco activities under maize-soybean intercropping (Oliveira et al., 2022).

The improved photosynthetic activities are directly associated with increased leaf area in response to N, Fe, and Mo application. This resulted in an improved light interception and subsequently higher carbon assimilation rates which agree with earlier findings (Wang et al., 2017; Nasar et al., 2021; Oliveira et al., 2022). In addition, as reported by Wang et al. (2017), the increase in photosynthetic activities could as well be linked with the upregulation of light-harvesting genes in photosystem II that improved net photosynthesis. Hussain et al. (2021) also reported that the increased leaf area of the soybean crop has significantly increased the photosynthetic activities of the soybean crop under maize-soybean intercropping, particularly under titanium application. These results are also in line with those of Derviş et al. (2018), who reported that the improved chlorophyll and photosynthetic activities are directly associated with an increase in leaf area. Moreover, the application of nitrogen, iron, and molybdenum application either alone or in combination are also reported earlier to increase the leaf area of the plant, thereby improving the chlorophyll and photosynthetic activities of the plant, which ultimately leads to higher crop yield (Vagusevičienė et al., 2013; Wang et al., 2017; Imran et al., 2019).

Changes in the photosynthetic capacity accompany a high quantity of changes in chlorophyll fluorescence parameters (i.e., Fv/Fm, øPSII, qp, and ETR) (Yao et al., 2017). Fv/Fm is used to measure the original light energy conversion efficiency of PSII in plant leaves. qp reflects the light energy absorbed and NPQ reflects the light energy dissipated in the form of heat whether the light energy is absorbed by PSII antenna pigment. This in turn is used to measure photosynthetic electron transfer (ETR) (Zhang et al., 2020; Tadmor et al., 2021; Martín-Clemente et al., 2022). Under environmental stress, the Fv/Fm, øPSII, qp, and ETR decreased and the NPQ increased, indicating that the ability of PS II to use light energy decreased, resulting in low electron transfer efficiency of carbon fixation, and the excess light energy lost in the form of heat dissipation.

The current study demonstrated that intercropping under _F0_ treatment reduced the Fv/Fm, øPSII, qp, and ETR, and increased the NPQ of the soybean crop. However, these indices of soybean crop under intercropping were increased in _F1_, _F2_, _F3_, and _F4_ treatments, but the _F4_ treatments showed more prominent results. The decreased leaf chlorophyll fluorescence parameters of soybean crops were due to the lower photosynthetic activities caused by the strong shading of maize crops in intercropping (Wang et al., 2017; Peng et al., 2021; Oliveira et al., 2022). However, the increase in leaf chlorophyll fluorescence parameters could be due to fertilizer application (i.e., N, Fe, and Mo). As reported earlier, nitrogen improves the photosynthetic activities and chlorophyll fluorescence of plants, because of its direct involvement in the component of chlorophyll content, enzyme content, and enzymatic activity (Qi et al., 2021). On the other hand, Fe is a key component of the ribulose 1,5 bisphosphate carboxylase/oxygenase, which is a key enzyme involved in plant photosynthesis (Wang et al., 2017) with Mo being directly or indirectly involved in the chlorophyll biosynthesis, chloroplast ultrastructure and rubisco enzymes (Imran et al., 2019). Rubisco is the predominant key enzyme protein involved in plant photosynthesis, contributing up to 20–30% of total leaf nitrogen and 50% of the total soluble leaf proteins (1^st^ primary source) (Lin et al., 2022). However, under environmental stress, plants showed a decrease in the rubisco enzymes, which causes a decline in the photosynthetic activities and soluble protein in plant leaves, thereby decreasing its yield (Feller, 2016; Shao et al., 2021; Rahimi et al., 2022; Taylor et al., 2022).

The study clearly shows that intercropping reduced the soluble protein content and rubisco activity of soybean at _F0_ treatment. However, the soluble protein and rubisco activity of intercropping soybean were significantly improved in _F1_< _F3_< _F2_< _F4_. The decrease in the Rubisco and soluble protein is linked to the shading effect under intercropping environment. However, the increase in rubisco activity and soluble protein could be due to the fertilizer application of N, Fe, and Mo as confirmed previously (Gai et al., 2017; Gülser et al., 2019; Oliveira et al., 2022). Taking together, the findings of this study suggested that optimal nitrogen fertilization combined with adequate foliar application of Fe and Mo can help improve the shade tolerance of soybean crops in maize-soybean intercropping by improving the chlorophyll, photosynthetic activities, and the associated enzyme, thereby enhancing its growth and yield.

## 5 Conclusions

Nitrogen fertilization combined with foliar application of iron and molybdenum compensated the shade induce lax growth by enhancing soybean agronomic parameters such as stem strength, stem diameter, and biomass dry matter in maize-soybean intercropping. The same fertilizers combination improved photosynthetic activities, chlorophyll content, and chlorophyll fluorescence of soybean crops by expanding leaf area and regulating the key enzymes and protein that had been damaged by shade stress under maize-soybean intercropping. As a result, nitrogen fertilization combined with foliar application of iron and molybdenum increases soybean yield under maize-soybean intercropping by up to 16 percent and therefore it’s worthy of being adopted by soybean growers.

## Data availability statement

The original contributions presented in the study are included in the article/supplementary material. Further inquiries can be directed to the corresponding author.

## Author contributions

Conceptualization, JN; Data curation, JN, GYW and IA; Formal analysis, FZ and MJ; Methodology, JN; Resources, XBZ and FZ; Software, JN; Supervision, XBZ; Writing – original draft, JN; Writing – review & editing, HG, KT, MH, HA and MW. All authors contributed to the article and approved the submitted version.

## Funding

This work was supported by the Natural Science Foundation of Guangxi Province (2019GXNSFAA185028).

## Acknowledgments

We acknowledge the Guangxi University, Nanning, for providing the experimental station and laboratory facility. We are also thankful to Prof. Dr. XBZ for his technical and advisory support throughout the study. The authors also like to thanks the Alexandria and University of Sadat for their technical support. We are also thankful to the Khwaja Fareed University of Engineering and technology, Pakistan and Sher-e-Bangla Agricultural University Dhaka Bangladesh for their moral support.

## Conflict of interest

The authors declare that the research was conducted in the absence of any commercial or financial relationships that could be construed as a potential conflict of interest.

## Publisher’s note

All claims expressed in this article are solely those of the authors and do not necessarily represent those of their affiliated organizations, or those of the publisher, the editors and the reviewers. Any product that may be evaluated in this article, or claim that may be made by its manufacturer, is not guaranteed or endorsed by the publisher.
